# The margination propensity of spherical particles for vascular targeting in the microcirculation

**DOI:** 10.1186/1477-3155-6-9

**Published:** 2008-08-15

**Authors:** Francesco Gentile, Antonio Curcio, Ciro Indolfi, Mauro Ferrari, Paolo Decuzzi

**Affiliations:** 1 Center of Bio-/Nanotechnology and -/Engineering for Medicine University of Magna Graecia at Catanzaro, Viale Europa – Loc. Germaneto, 88100, Catanzaro, Italy; 2 Division of Cardiology, University of Magna Graecia at Catanzaro Viale Europa – Loc. Germaneto, 88100, Catanzaro, Italy; 3 The University of Texas Health Science Center Houston 1825 Pressler St, Houston, Texas, 77030, USA; 4 M.D. Anderson Cancer Center and Rice University 1825 Pressler St, Houston, Texas, 77030, USA

## Abstract

The propensity of circulating particles to drift laterally towards the vessel walls (margination) in the microcirculation has been experimentally studied using a parallel plate flow chamber. Fluorescent polystyrene particles, with a relative density to water of just 50 *g/cm*^3^comparable with that of liposomal or polymeric nanoparticles used in drug delivery and bio-imaging, have been used with a diameter spanning over three order of magnitudes from 50 *nm *up to 10 *μm*. The number ${\tilde{n}}_{s}$ of particles marginating per unit surface have been measured through confocal fluorescent microscopy for a horizontal chamber, and the corresponding total volume ${\tilde{V}}_{s}$ of particles has been calculated. Scaling laws have been derived as a function of the particle diameter *d*. In horizontal capillaries, margination is mainly due to the gravitational force for particles with *d *> 200 *nm *and ${\tilde{V}}_{s}$ increases with *d*^4^; whereas for smaller particles ${\tilde{V}}_{s}$ increases with *d*^3^. In vertical capillaries, since the particles are heavier than the fluid they would tend to marginate towards the walls in downward flows and towards the center in upward flows, with ${\tilde{V}}_{s}$ increasing with *d*^9/2^. However, the margination in vertical capillaries is predicted to be much smaller than in horizontal capillaries. These results suggest that, for particles circulating in an external field of volume forces (gravitation or magnetic), the strategy of using larger particles designed to marginate and adhere firmly to the vascular walls under flow could be more effective than that of using particles sufficiently small (*d *< 200 *nm*) to hopefully cross a discontinuous endothelium.

## 1 Introduction

In the early diagnosis, treatment and imaging of diseases, as cancer and cardiovascular, the use of microparticles and nanoparticles is emerging as a powerful tool [1,2]. These are sufficiently small 'vectors' of therapeutic or/and imaging agents to be systemically administered, transported by the blood flow along the circulatory system and eventually recognize the diseased microenvironment (diseased cells). A nanoparticle comprises an internal core with the active agents and an external coating whit tailored physico-chemical properties. The interaction of the vectors with the biological target (diseased cell) is generally governed by specific forces, mediated by the formation and destruction of molecular bonds [3], and by non-specific interactions regulated by short ranged forces as van der Waals, electrostatic and steric [4].

Two different delivery strategies are currently under investigation and development: *a passive targeting of the diseased microenvironment *relying on the permeability of the blood vessels (*enhanced retention and permeability effect*), and an *active targeting of the diseased microvasculature *relying on the recognition of specific molecules overexpressed at the site of interest [5]. It is known that tumor microvessels exhibit a significant increase in permeability to large molecules with intercellular openings and intercellular gaps as large as a micron [6], which could be crossed by sufficiently small particles. However the level of permeability is strongly dependent on the type of tumor, the site where the tumor is developing, the state of the tumor and the therapeutic treatment, and significant differences can be observed between human and xenografts tumors [7]. In addition to this, diseases other than cancer do no show any significant vessel permeability, thus making a passive targeting strategy non appropriate. On the other hand, a growing body of evidences support the idea that specific molecules are overexpressed at the surface of a diseased vasculature [8], which could be used as 'docking sites' for circulating particles. Following a microvas-culature targeting strategy could possibly be more effective than just relying on the matching between the size of the particles and that of the vascular fenestrations. Evidently, the specific recognition and firm adhesion of a circulating particle to the vessel walls under flow is far from being an easy task.

For both delivery strategies, the systemically administered particles should be designed to move in close proximity to the vascular walls, 'sense' any significant biological difference between normal and abnormal endothelium and seek for fenestrations in the case of a passive strategy, or for specific vascular receptors, in the case of an active targeting strategy. In other words, the nanoparticles should be designed to spontaneously 'marginate', i.e. drift laterally towards the vessel walls, and interact with the blood vessels rather than 'navigating' in the center of the capillary as red blood cells (RBCs) do or, even worse, adhering and being transported by the RBCs. It is here important to recall that in physiology the term margination is referred to the lateral drifting of leukocytes which have been observed experimentally to collect near the walls of blood vessels. This behavior has been mainly associated to the interaction of leukocytes with RBCs which tend to push the former away from the center of the capillary towards the opposing wall [9], and it is no at all related to gravitational forces, as clearly demonstrated by [10]. Systemically administered particles for the delivery of drugs and other therapeutic agents have a characteristic size at least one order of magnitude smaller than leukocytes and RBCs (*O *(10) *μm*), and, more importantly, than the thickness of the cell free layer (*O *(10) *μm*). As a consequence, the margination of nanoparticles can not only rely on the interaction with other circulating cells, especially in the microcirculation where RBCs are less abundant. The margination dynamics of nanoparticles has to be controlled by their size, their shape and their possible interaction with external long range force fields, as the gravitational and electromagnetic fields.

In this work, the propensity to marginate of classical spherical particles in a laminar flow and under the effect of gravitational forces is studied. Particles with different diameters spanning from 50 *nm *up to 10 *μm *are infused within a parallel plate flow chamber mimicking the physiological conditions of human microcirculation. The density of the particles relative to water is of just 50 *g/cm*^3^, comparable with that of liposomal and polymeric based particles used in such applications.

## 2 Materials and methods

### The Flow Chamber System

**Figure 1:**
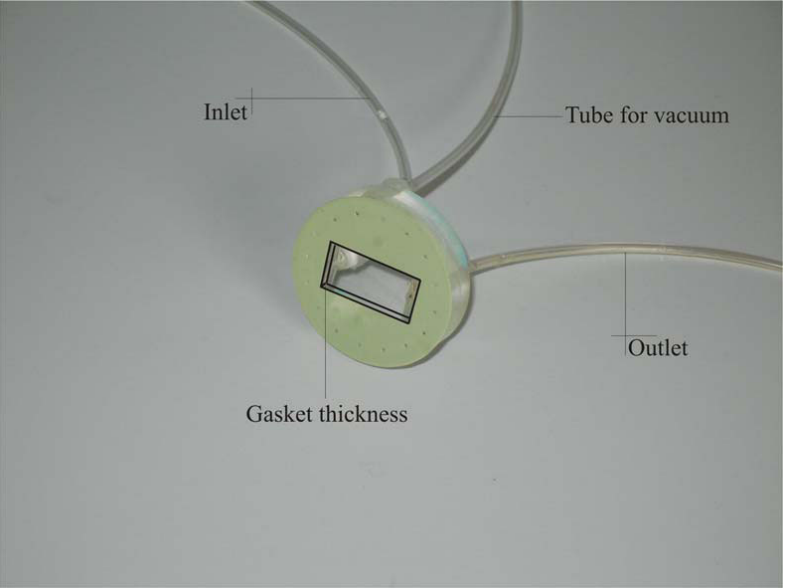
The PMMA flow deck of the flow chamber with the inlet and outlet tubings, and the gasket with a thickness of 254 *μm*.

The flow chamber system consists in a parallel plate flow chamber (from Glycotech – Rockville, MD) installed upon a 35 *mm *cell culture dish, where the particles are injected by means of a Harvard Apparatus syringe pump. The chamber is made up of a PMMA flow deck with an inlet and an outlet holes connected through silastic tubing to a syringe pump and a reservoir respectively (Fig. 1). Sitting between the chamber and the dish, a silicon rubber gasket defines the geometry of the channel where the particle solution is introduced. The gasket used in the present experiments has a thickness *h *of 254 *μm *(0. 01 *in*), a width *w *of 1 *cm *and a length *L *of 2 *cm*. The volumetric flow rate *Q *defined through the syringe pump has been fixed equal to 50 *μl/min *for all the experiments. Based on these data, the mean velocity *U *(= *Q*/(*wh*)) within the chamber is of about 0. 328 *mm/s*, a physiologically relevant value for human capillaries; the shear rate *S *at the substrate is given by the commonly used relation

(1) *S *= 6*Q*/*h*^2^*w *= 7.75 *s*^-1^

and the shear stress at the wall *ηS *= 7. 75 × 10^-3 ^*Pa *being *η *= 10^-3 ^*Pa s *the viscosity of water. The channel Reynolds number (= *ρUh*/*η*) is equal to about 8 × 10^-2^. The shear rate and the shear stress are sufficiently small to allow for the non-specific cell-particle adhesion. Experiments were performed at room temperature (24°*C*) for a maximum time of 10 min.

### The Measurement Set-up

**Figure 2:**
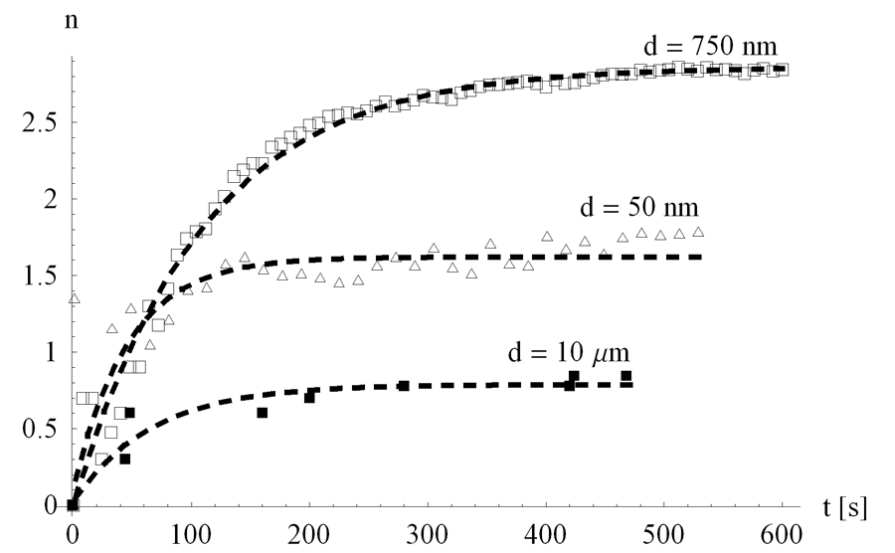
The number n × 10^3 ^of marginating particles as a function of time during a typical flow chamber experiment for three different particle sizes (*d *= 50 *nm*; 750 *nm *and 10 *μm*).

The flow chamber was mounted on the stage of a Leica TCS-SP2^® ^laser scanning confocal microscope system provided with a DM-IRB inverted microscope. The cells and the particles in the chamber were imaged using a 20× dry microscope objective – a field of view comprising 325 × 325 *μm*^2 ^was mapped into 256 × 256 lines, a resolution allowing for a line frequency of 800 *Hz*, and an acquisition rate derived as 800/256 ≃ 3 *fps*, which is fast enough to record continuously the dynamics of the particles. The pinhole (~80 *μm*) and laser power (argon/krypton: 80% power) were maintained throughout each experiment. Confocal images of green fluorescence were collected using a 488 *nm *excitation light. Both the Bright Field images of the cell substrate and the fluorescent confocal images of the nanoparticles were exported as tiff files into MatLAB^® ^and Mathematica^® ^where were deconvoluted using in-house developed software. The number of particles adhering to the whole substrate within the region of interest and the number of particles adherent to the sole cells within the region of interest were monitored for the whole duration of the experiment. Fig. 2 presents the total number of particles adhering within the region of interest for three different particles sizes.

### Cell Culture

Human umbilical vein endothelial cells (HUVECs) were purchased from Cambrex, Inc. (East Rutherford, NJ). Cells were maintained in EGMTM-2 – Endothelial Cell Medium-2 (Cambrex Bio Science Walkersville Inc., MD) supplemented with 2% FBS, 0. 04% hydrocortisone, 0. 4% hFGF-B, 0. 1% VEGF, 0. 1% rIGF-1, 0. 1% ascorbic acid, 0. 1% hEGF, 0. 1% GA-1000, 0. 1% heparin, 100 *U/mL *penicillin, and 100 *μg/mL *streptomycin and were grown at 37°*C *with humidified 95% air/5% *CO*2.

For each experiment, cells were plated on a borosilicate glass with a 0. 2 *mg/cm*^2 ^substratum of type A gelatine (Sigma-Aldrich Corporation, MO). When HUVECs reached 80% confluence, the borosilicate glass was detached from the bottom of the plate and mounted in the parallel plate flow chamber for particle-cell adhesion analysis.

### The Particles

Fluoresbrite^® ^Microspheres from Polysciences were used. These are Yellow Green fluorescent particles with an excitation maximum at 441 *nm *and an emission maximum at 486 *nm*. Particles with different sizes were used namely 50 *nm*, 100 *nm*, 200 *nm*, 500 *nm*, 750 *nm*, and 1 *μm*, 6 *μm*, 10 *μm*.

## 3 Results and discussions

The lateral drifting of particulates in capillary flow has been analyzed since the pioneering experiments of Poisuille in 1836 [11] who observed that RBCs do not distribute uniformly leaving a region devoid of particles in close proximity to the walls: the cell free layer. More recently, Segré and Silberberg [12] have showed that for small Reynolds numbers a bolus of neutrally-buoyant particles would preferentially migrate towards the walls of the tube leading to a non uniform radial distribution with a peak at about 0. 6 times the capillary radius. After Segré, many authors experimentally investigated the behavior of non neutrally buoyant rigid spheres [13-16], finding out that the equilibrium position would depend on the relative density of the particles to the fluid and the flow Reynolds number. In 1994, Hogg [17] has presented a comprehensive theoretical analysis for the migration of non-neutrally buoyant spherical particles in two-dimensional shear flows. Three different dimensionless parameters have been introduced to describe the problem: the geometric ratio *α *(= *d*/(2*h*)) between the particle diameter *d *and the channel height *h*; the channel Reynolds number *Re*_*c *_(= *ρ*_*f*_*U*_*m*_*R*_*h*_/*μ*) with *ρ*_*f *_the density, *μ *the viscosity and *U*_*m *_the mean flow velocity of the fluid, and the hydraulic radius *R*_*h *_of the channel (*R*_*h *_= 2*hw*/(*h *+ *w*)); and the buoyancy number *B *= *d*^2 ^Δ_*ρg*_/(18 *μU*_*m*_), being Δ*ρ *the density of the particle relative to the fluid. Different marginating behaviors have been identified depending on the values of the combined parameters *α*^2^/B and *Re*_*c*_*B*^2 ^compared to unity. In the microcirculation, with *U*_*m *_of *O *(100) *μm/s*, *h *and *R*_*h *_of *O *(100) *μm*, and for *d *of *O *(10) *μm *and smaller, it follows

$$\begin{array}{ccc} \alpha^{2}/B=\frac{9}{2}\frac{\mu U_{m}}{h^{2}∆\rho g}<O\;(10^{-2}) & \text{and} & Re_{c}B^{2}=\frac{1}{18}R_{h}d^{2}\frac{\rho_{f}∆\rho g}{\mu^{2}}<O\;(10^{-1}) \end{array}$$

having considered *μ *= 10^-3 ^*Pa s*, Δ*ρ *= 50 *kg/m*^3 ^and *ρ*_*f *_= 10^3 ^*kg/m*^3^. For the flow chamber apparatus considered here and *d *= 10 *μm*, it is *α *≃ 0. 04, *Re*_*c *_≃ 0. 16 and *B *≃ 0. 053 leading to *α*^2^/*B *≃ 0. 03 and *Re*_*c*_*B*^2 ^≃ 4. 6 × 10^4^, much smaller than unity.

### 3.1 Margination in Horizontal Capillaries

**Figure 3:**
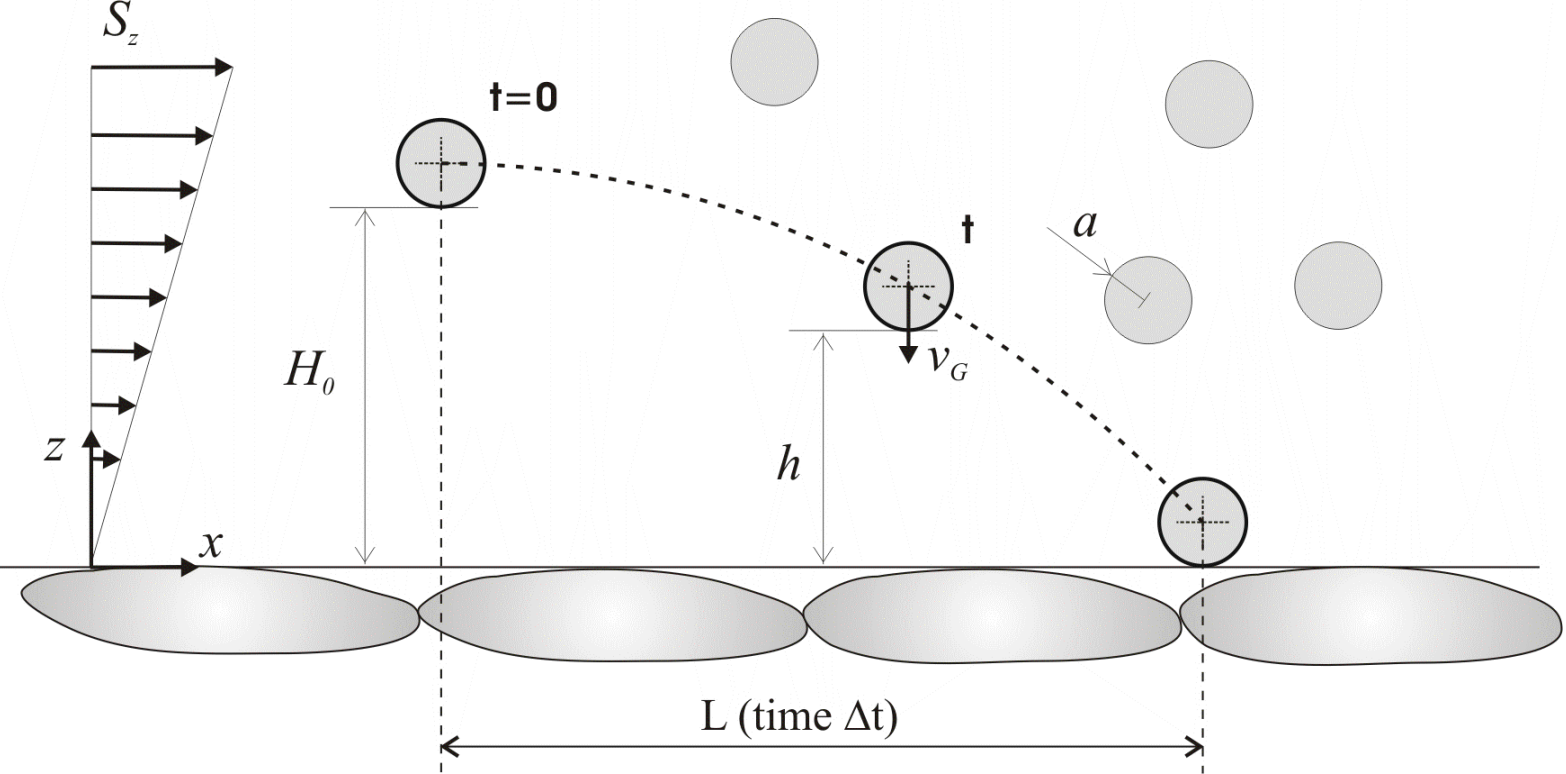
The margination trajectory of a spherical particle within a laminar flow.

For *α*^2^/*B *< 1 and *Re*_*c*_*B*^2 ^< 1, as observed in [17], the lateral drift is mainly due to the gravitational force acting orthogonally to the flow direction and the drifting velocity is only slightly different from that predicted by Stokes for the settling of a particle in a quiescent fluid, that is to say

(2) $$v^{G}=\frac{d〈h〉}{dt}=\frac{1}{18}\frac{g∆\rho }{\mu }d^{2}=BU_{m}$$

where ⟨*h*⟩ is the average separation distance of the particle from the wall (see Fig. 3). Integrating (2) over an initial separation distance ⟨*H*_*o*_⟩, and observing that when at ⟨*H*_*o*_⟩ the particle would move longitudinally by the distance *L *in a time Δ*t *= *L*/(S⟨*H*_*o*_⟩), it follows that

(3) $${\left\langle H_{o}\right\rangle }^{2}=\left[\frac{1}{18}\frac{g∆\rho }{\mu }\frac{L}{S}\right]\times d^{2}$$

where *L *is the length of the region of interest with surface area *A*. Notice that in the case of a magnetic particle driven towards the chamber substrate by an external magnetic field, ⟨*H*_*o*_⟩ would scale with *d *as in (3), being the magnetic force *F*_*mag *_proportional to the volume of the particle, just as the gravitation force. The separation distance ⟨*H*_*o*_⟩ multiplied by *A *gives the volume of fluid within which are comprised the particles candidate to sediment within the region of interest (the sedimentation volume). If *C *is the local concentration of the particles within the sedimentation volume, the number of depositing particles per unit area *A *is readily given by

(4) $${\tilde{n}}_{s}=\frac{CV_{s}}{A}=C\langle H_{o}\rangle \propto C\times d$$

whereas the total volume of settling particles ${\tilde{V}}_{s}$ per unit surface is defined as

(5) $${\tilde{V}}_{s}=C\langle H_{o}\rangle \times \pi d^{3}/6\propto C\times d^{4}$$

Substituting in (4) and (5) for (3), it follows that under a gravitational field (or magnetic field) the number ${\tilde{n}}_{s}$ of settling particles per unit area and their volume ${\tilde{V}}_{s}$ are both proportional to the local volume concentration *C *of particles and grows linearly with *d *(${\tilde{n}}_{s}$ ∝ *d*) and with the fourth power of *d *(${\tilde{V}}_{s}$ ∝ *d*^4^), respectively.

**Figure 4:**
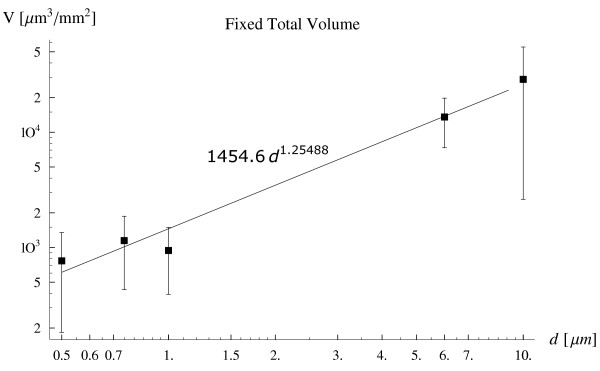
The volume of particles marginating per unit surface ${\tilde{V}}_{s}$ as a function of the particle diameter *d *ranging from 500 *nm *up to 10 *μm *(fixed total volume of the injected particles *V*_*tot *_= 5.2 × 10^7 ^*μm *^3 ^and *C*_*v *_= 5.2 × 10^5^).

**Figure 5:**
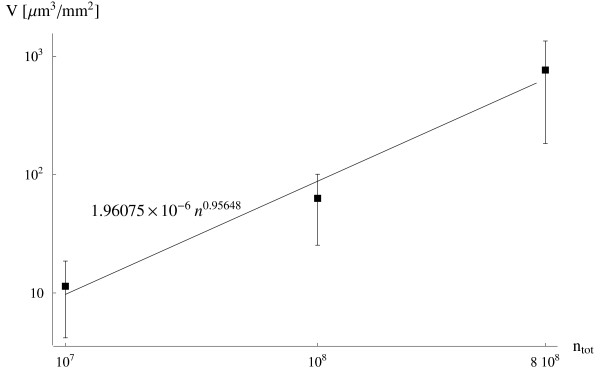
The volume of particles marginating per unit surface ${\tilde{V}}_{s}$ as a function of the particle total number *n*_*tot *_(fixed diameter *d *= 500 *nm*).

The above 'back of the envelope' calculations, although extremely simplified, are in decent agreement with the experimental results obtained using the parallel plate flow chamber apparatus. The total volume of the particles settling per unit surface ${\tilde{V}}_{s}$ is shown as a function of the particle diameter in Fig. 4, ranging between 500 *nm *and 10 *μm*. These experiments have been performed keeping fixed the total volume of the particles injected into the chamber (i.e. 5. 2 × 10^7 ^*μm*^3^/*ml*) for each particle size, or in other words, the total number of particles injected decreases with *d*^-3. ^In a double-logarithm diagram, the average values of ${\tilde{V}}_{s}$ over five significant repetitions (filled boxes) are well aligned around a straight line with a nearly unit slope described by the relation

(6) $$\begin{array}{ccc} {\tilde{V}}_{s}=1454.6\;d^{1.2}, & \text{with} & R^{2}=0.976 \end{array}$$

which gives almost the same scaling as predicted in (5), assuming a fixed total volume of injected particles. In Fig. 5, the variation of the volume ${\tilde{V}}_{s}$ as a function of the total number of particles injected *n*_*tot *_in the flow chamber is plotted for a fixed particle size (*d *= 500 *nm*), showing a linear increase of ${\tilde{V}}_{s}$ following the experimental relationship

(7) $$\begin{array}{ccc} {\tilde{V}}_{s}=196\times 10^{-6}n_{tot}^{0.956}, & \text{with} & R^{2}=0.981 \end{array}$$

which support the linear relationship between ${\tilde{V}}_{s}$ and *C *as predicted in (5).

**Figure 6:**
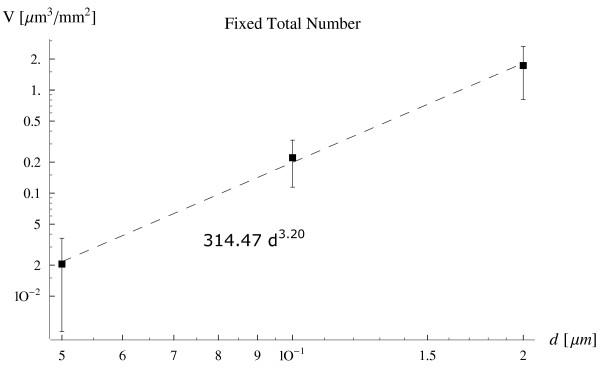
The volume of particles marginating per unit surface ${\tilde{V}}_{s}$ as a function of the particle diameter *d *ranging from 50 to 200 *nm *(fixed total number of the injected particles *n*_*tot *_= 10^8 ^and *C *= 10^14 ^*m*^-3^).

In Fig. 6, the behavior of the smaller particles is considered with a diameter ranging from 200 *nm *down to 50 *nm*. However, differently from the previous case, shown in Fig. 4, the analysis has been performed for a fixed number of injected particles (*n*_*tot *_= 10^8^), to limit the total amount of particles to be used for a diameter of 50 *nm*. The ${\tilde{V}}_{s}$ - *d *relation is different from that observed for the larger particles, and it is no more nearly linear in a double-logarithm diagram being

(8) $$\begin{array}{ccc} {\tilde{V}}_{s}=314.5\;d^{3.2}, & \text{with} & R^{2}=0.998 \end{array}$$

whose scaling with *d *can not be predicted by just gravitation or volume forces. For these small particles other forces as colloidal forces (van der Waals, electrostatic) are probably responsible for their margination, which arise only with a small separation distance between the particle and the substrate (tens to a hundred nanometers). An ANOVA analysis has returned, for the data presented in Fig. 4 to 6, *p *values much smaller than the critical value of 0. 05, being respectively *p *= 0. 0022, *p *= 0. 0035, and *p *= 0. 0047, thus implying a statistically significant difference among the means.

### 3.2 Margination in Vertical Capillaries

For *α*^2^/*B *and *Re*_*c*_*B*^2 ^smaller than unity, as observed in [17], in vertical capillaries the lateral drift is modest with a velocity scaling with $BR_{p}^{1/2}$, where *R*_*p *_is the particle Reynolds number (*R*_*p *_= *ρ*_*p*_*U*_*m*_*d*^2^/(*μR*_*ch*_)). Therefore the lateral drifting velocity would scale with *d*^3^rather with *d*^2 ^as in horizontal capillaries (see eq.2), making the characteristic size of the particles even more important. Following the same reasonings as above for the horizontal capillaries, it can be derived a ⟨*H*_*o*_⟩ scaling with *d*^3/2^, and eventually a number ${\tilde{n}}_{s}$ and a volume ${\tilde{V}}_{s}$ of settling particles per unit area proportional to the local volume concentration *C *of particles and scaling respectively with *d*^3/2 ^and *d*^9/2 ^(${\tilde{n}}_{s}$ ∝ *d*^3/2 ^and ${\tilde{V}}_{s}$ ∝ *d*^9/2^).

The lateral drifting observed in vertical capillaries is again associated with the difference in relative density between the circulating particle and the fluid, being *B*, the buoyancy parameter, different from zero. But more importantly, the sign of the velocity depends on the direction of the flow: particles heavier than the fluid would drift towards the wall for downward flows (margination) and towards the capillary center line for upward flows (opposite of margination). The opposite has been predicted and observed to occur for particles less heavy than the fluid. These behavior has been observed extensively in several experiments [18].

## 4 Conclusion

The propensity of spherical nanoparticles to marginate towards the vessel walls in the microcirculation has been analyzed employing a parallel plate flow chamber. The effect of the particle size and orientation of the capillary with respect to external volume force fields (gravitation) has been elucidated experimentally and supported by simple scaling relations.

The number ${\tilde{n}}_{s}$ and total volume ${\tilde{V}}_{s}$ of particles marginating per unit surface have been measured through confocal fluorescent microscopy. Considering particles with a density slightly larger than water (1050 *kg/m*^3^), and comparable with the density of liposomes and polymeric particles used in nanomedical applications, it has been observed in horizontal channels that the lateral margination of particles with a diameter larger than 200 *nm *is mainly governed by the gravitational force with ${\tilde{n}}_{s}$ and ${\tilde{V}}_{s}$ scaling both proportionally to the volume concentration *C *of the particles and, respectively, to the diameter *d *and the fourth power of the diameter *d*^4^. For smaller particles (*d *< 200 *nm*), the margination dynamics can not be associated to gravitational forces being ${\tilde{V}}_{s}$ ∝ *d*^3.2^. Possibly, in this case, colloidal interactions may govern particle lateral drifting but this would already require the particle to be in sufficient close proximity of the wall, say tens up to a hundred nanometer, in other words separation distances of the same order of magnitude of the particle size.

These results, although not exhaustive, are of interest in the systemic delivery of nanoparticles designed to target the vascular walls in the microcirculation. The experimental results and simple theoretical relations support the idea of using large particles rather than small particles with the same total volume. In fact, if the biological target is the vascular wall and the particles are not required to freely extravasate through the discontinuous endothelium, then the larger spherical particles would more easily sediment in horizontal capillaries and drift laterally in vertical capillaries with downward flow. Also the larger spherical particles would have a larger surface exposed to the vascular cells increasing the likelihood of firm adhesion once decorated with recognizing moieties [3]. The separation between large and small particles would depend on the relative density compared to the fluid, however for the commonly used liposome and polymeric particles sizes larger than 200 *nm *would perform better.

It should be noticed, in conclusion, that the present results strictly apply when the interaction of the nanoparticles with circulating blood cells can be disregarded, which occurs in small capillaries and in the cell free layer of arterioles and veins.
